# Weak Interactions of the Isomers of Phototrexate and Two Cavitand Derivatives

**DOI:** 10.3390/ijms221910764

**Published:** 2021-10-05

**Authors:** Zsolt Preisz, Zoltán Nagymihály, László Kollár, Tamás Kálai, Sándor Kunsági-Máté

**Affiliations:** 1 Faculty of Pharmacy, Institute of Organic and Medicinal Chemistry, University of Pécs, Szigeti 12, 7624 Pécs, Hungary; preisz.zsolt@pte.hu (Z.P.); tamas.kalai@aok.pte.hu (T.K.); 2 Department of Physical Chemistry and Materials Science, Faculty of Sciences, University of Pécs, Ifjúság 6, 7624 Pécs, Hungary; 3 János Szentágothai Research Center, University of Pécs, Ifjúság 20, 7624 Pécs, Hungary; nmzoltan@gamma.ttk.pte.hu (Z.N.); kollar@gamma.ttk.pte.hu (L.K.); 4 Department of Inorganic Chemistry, Faculty of Sciences, University of Pécs, Ifjúság 6, 7624 Pécs, Hungary

**Keywords:** Phototrexate, cavitand, chemotherapy, inclusion complex, thermodynamics, fluorescence

## Abstract

The interactions of two conformers of newly synthesized photoswitchable azobenzene analogue of methotrexate, called Phototrexate, with two cavitand derivatives, have been investigated in dimethyl sulfoxide medium. Photoluminescence methods have been applied to determine the complex stabilities and the related enthalpy and entropy changes associated to the complex formation around room temperature. Results show opposite temperature dependence of complex stabilities. The structure of the upper rims of the host molecules and the reordered solvent structure were identified as the background of the opposite tendencies of temperature dependence at molecular level. These results can support the therapeutic application of the photoswitchable phototrexate, because the formation of inclusion complexes is a promising method to regulate the pharmacokinetics of drug molecules.

## 1. Introduction

Cancer is among the leading causes of death worldwide. Before the 1940s, only surgical therapy existed to treat cancer. It was demonstrated for the first time in 1942 that chemotherapy can induce tumor regression [1]. In the late 1940s, it was found that antimetabolites that inhibit the function of folate-requiring enzymes can induce remission in children with acute lymphoblastic leukaemia. These antimetabolites are the inhibitors of the enzyme dihydrofolate reductase (DHFR) and cause decreasing thymidylate synthesis which ultimately inhibits DNA synthesis [1].

**Figure 1:**
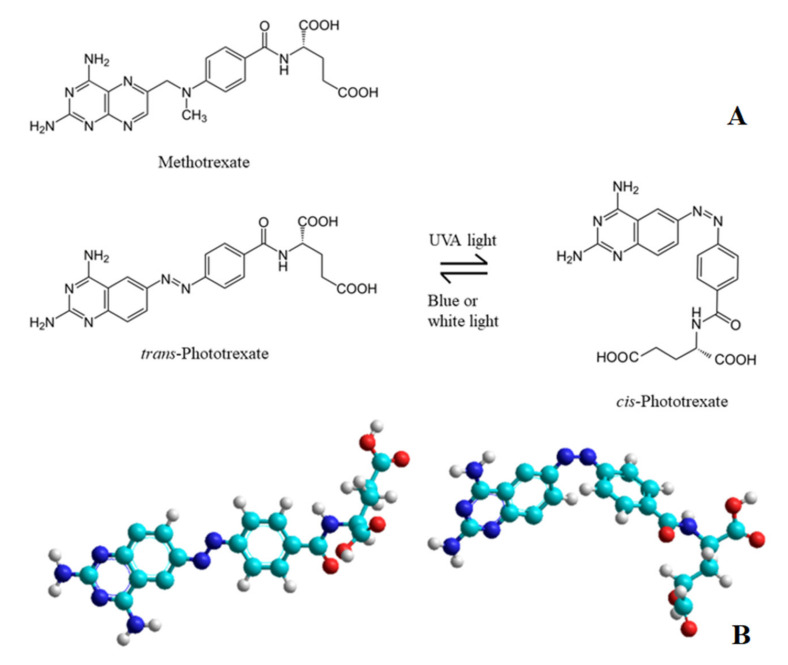
(**A**) Chemical structures of Methotrexate (MTX), *trans*-Phototrexate (*trans*-PHX) and *cis*-Phototrexate (*cis*-PHX) and the reversible isomerization of PHX [11]. (**B**) The 3D structure of the two isomers of the PHX: *trans*-PHX (**left**) and *cis*-PHX (**right**).

Methotrexate (4-amino-10-methylfolic acid, MTX, Figure 1) is the most important antimetabolite chemotherapeutic agent, it is primarily used in acute lymphoblastic leukaemia, in certain lymphomas, osteosarcoma and choriocarcinoma [1]. In the 1980s, it was discovered that, at low doses, MTX has “steroid-like” anti-inflammatory and immunosuppressant effects and, in 1985, it was clinically demonstrated to be a potent and effective treatment in psoriatic arthritis and rheumatoid arthritis, so, nowadays, it is a first line drug for these autoimmune diseases [2].

However, MTX and other chemotherapeutic agents can cause several adverse effects, because they affect every fast dividing cell of the body. Some toxicities are unrelated to folate antagonism and cannot be prevented by folate supplementation. These include nodulosis, hepatic fibrosis, pulmonary fibrosis, lethargy, fatigue, and renal insufficiency [3]. Association has been found between MTX use and increased risk for melanoma, lung cancer and non-Hodgkin lymphoma [4].

Alternative strategies to overcome low therapeutic indices, nonspecific targeting, and the off target toxicity of chemotherapeutics have emerged over the last years [5,6]. One interesting and recent research field is photopharmacology. The principle of photopharmacology is the introduction of a photoswitchable unit into the molecular structure of a bioactive compound itself [7]. One major advantage of photopharmacological agents is that their activation is reversible, which may lead to a significant reduction in adverse drug reactions [8]. In general, this strategy uses molecules that can be efficiently excited to the more active form and spontaneously reisomerize to the less active isomer. The biological functions of photopharmacological molecules can be controlled with synthetic photoswitches. Photoswitches are chromophores that can be reversibly isomerized when exposed to light. Azobenzene is the most widely used photoswitch in biological applications because of the ease of synthesis and functionalization, fast photoisomerization, and low rate of photobleaching [9,10]. 

Phototrexate (PHX, Figure 1) is a photoswitchable azobenzene analogue of MTX that has been synthesized and described by Matera et al. [11] and by Mashita et al. [12]. PHX contains a diazene stereogenic unit and its pharmacological activity is higher in its *cis* state than in the thermodynamically more stable *trans* state. It can be effectively isomerized from *trans* to *cis* with UVA light and isomerized back from *cis* to *trans* with blue or white light (Figure 1). This transition is reversible and can be repeated several times. The antineoplastic effect (and, also, the adverse effects caused by the cytotoxic activity) appears only in light exposed regions and decreases in dark regions [11]. Target tissues might be those that can be exposed to UV illumination, primarily the skin, the digestive, respiratory and reproductive tracts.

Calixarenes are cyclic oligomer molecules that consist of phenolic units that are condensated in the presence of an aldehyde and linked to each other, typically through a methylene bridge, in an acidic environment [13,14]. They are widely used as host molecules in supramolecular chemistry, separation science and catalysis. They also have pharmaceutical applications as host molecules [14]. Substituents appended to the phenolic rings (upper or lower rim) can greatly influence the physical and chemical properties of these molecules, but numerous derivatives have been synthesized that have functional groups on the periphery of the molecule, too [15]. Promising results have been described in the literature about calixarenes that have heterocyclic substituents on the periphery, they can be used as selective extractants for amino acids [16], chiral recognition agents [17] or chelators that trap metals [18]. Calix[4]arene-based P-ligands were used in rhodium-catalyzed hydroformylation [19]. Inserting additional methylene bridges between the phenolic oxygen atoms on adjacent aromatic rings, the structure is called a cavitand.

**Figure 2:**
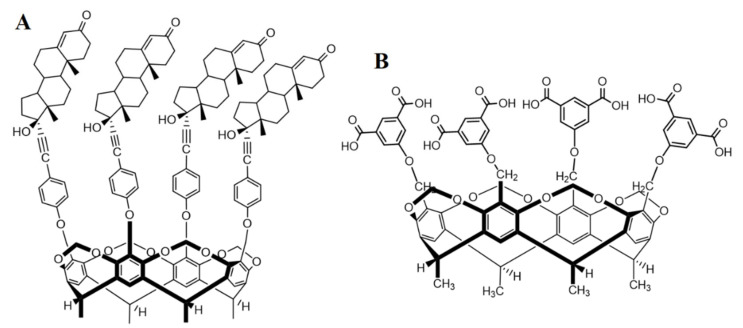
Chemical structures of the macrocyclic host molecules TAC (**A**) and TDC (**B**) (8-H, 9-H and 13-H of the steroidal skeleton are omitted for clarity).

No study has been found in the literature that describes the host–guest complex formation or any weak molecular interaction of PHX, so cavitand–PHX interactions are an entirely unrevealed research field. Therefore, in this work, the thermodynamic parameters of the interaction of PHX with tetrakis(androst-4-en-3-one-17α-ethinyl)-cavitand (TAC) and tetrakis(3,5-dicarboxylatophenoxy)-cavitand (TDC) (Figure 2) were studied. TAC was synthesized as previously described [20]. The synthesis of TDC is based on the Williamson-type ether synthesis, reacting tetrabromo-cavitand [21,22] with dimethyl 5-hydroxyisophthalate, followed by the hydrolysis of the ester functionalities. Fluorimetric measurements were applied to determine the thermodynamic parameters of the PHX–cavitand complex formation reaction. 

## 2. Results

### 2.1. Determination of the Association Constants

To investigate the interaction of *cis*-PHX with the cavitand derivatives (TAC and TDC), the complete isomerization of the thermodynamically more stable *trans*-PHX has to be reached. To this purpose, UV light exposure (λ = 366 nm) was used, as in our previous work [23].

**Figure 3:**
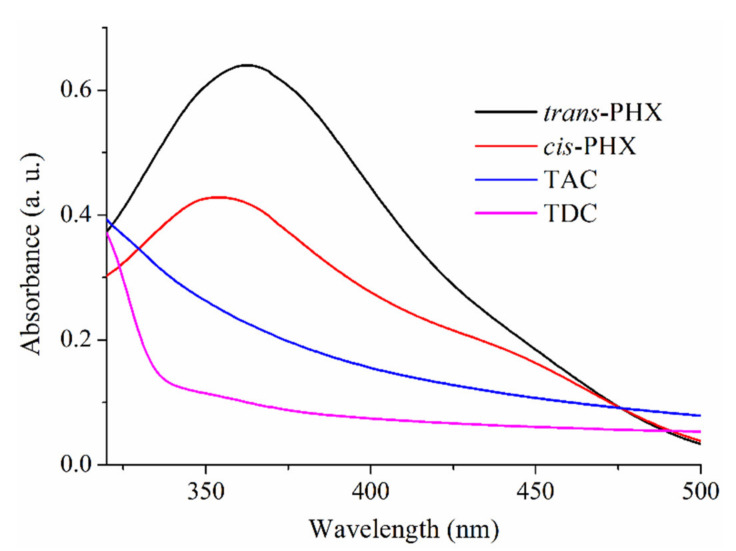
Absorption spectra of *trans*-PHX, *cis*-PHX, TAC and TDC at 50 µM concentration.

The absorption spectra of the isomers of PHX and the cavitand derivatives were registered (Figure 3). The absorption maxima of *cis*- and *trans*-PHX were at around 370 nm, while TDC and TAC showed significantly lower absorption values at this wavelength. Accordingly, applying a 366 nm excitation wavelength, the emission spectra of PHX were recorded. 

**Figure 4:**
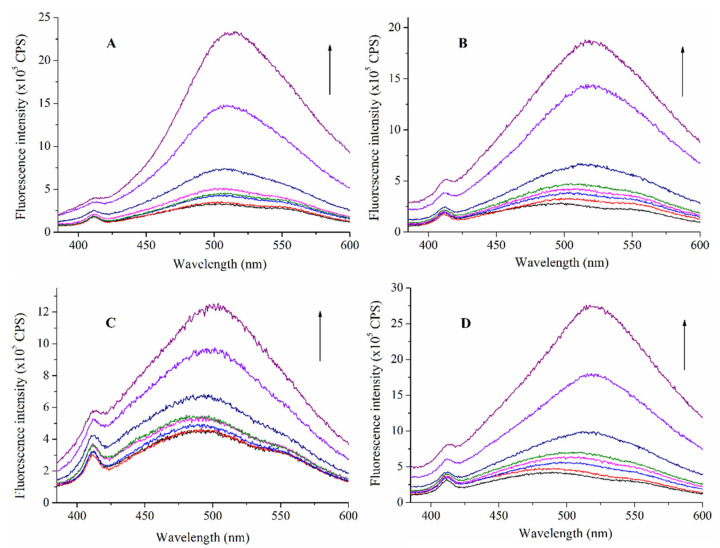
Fluorescence emission spectra of *trans*-PHX (**A**,**B**) and *cis*-PHX (**C**,**D**) (50 µM) in the absence and presence of TAC (**A**,**C**) and TDC (**B**,**D**) (0–450 µM) (λ_exc_ = 366 nm). The figure depicts the measurements that were carried out at 293 K. Arrows indicate increasing cavitand concentrations. Colors only support the clarity.

The fluorescence spectra of *cis*- and *trans*-PHX show increased emission upon increased concentration of TAC or TDC (Figure 4). The changes induced in the spectra of PHX suggest interactions between the investigated molecules. Samples with different concentrations of the cavitand derivates (0–450 μM) and with constant concentration of *trans*- and *cis*-PHX (50 μM) were prepared and measured using a 366 nm excitation wavelength at 293 K, 298 K, 303 K and 308 K temperatures. 

The stability constants of the *trans*-PHX-TAC, *trans*-PHX-TDC, *cis*-PHX-TAC and *cis*-PHX-TDC have been determined using the Benesi–Hildebrand method. To determine the thermodynamic parameters, the stability constants have been calculated at temperatures 293 K, 298 K, 303 K and 308 K. In all cases, the intensity values obtained at 490 nm have been used to evaluate the data. Table 1 summarizes the results. The determined stability constants were then used to calculate the thermodynamic parameters (ΔH, ΔS, and ΔG) of the interactions (Table 2).

**Figure 5:**
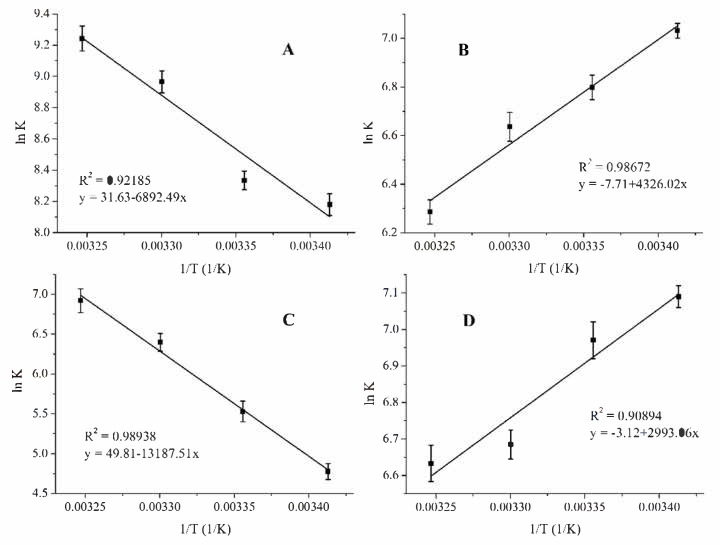
Van ‘t Hoff plots of the complex formation of *trans*-PHX and TAC (**A**), *trans*-PHX and TDC (**B**), *cis*-PHX and TAC (**C**) and *cis*-PHX and TDC (**D**).

The Van ‘t Hoff plots of the investigated interactions have been created by plotting the logarithms of the stability constants against the reciprocal temperatures (Figure 5). It can be observed that the thermodynamic parameters of the complex formation of the two cavitand derivates differ significantly. 

These results imply different complex formation mechanisms for the different cavitand derivates (TAC and TDC). In the presence of TAC, entropy gain is associated with enthalpy gain, while in the samples containing TDC, entropy loss is associated with enthalpy loss. 

### 2.2. Modelling Studies

**Figure 6:**
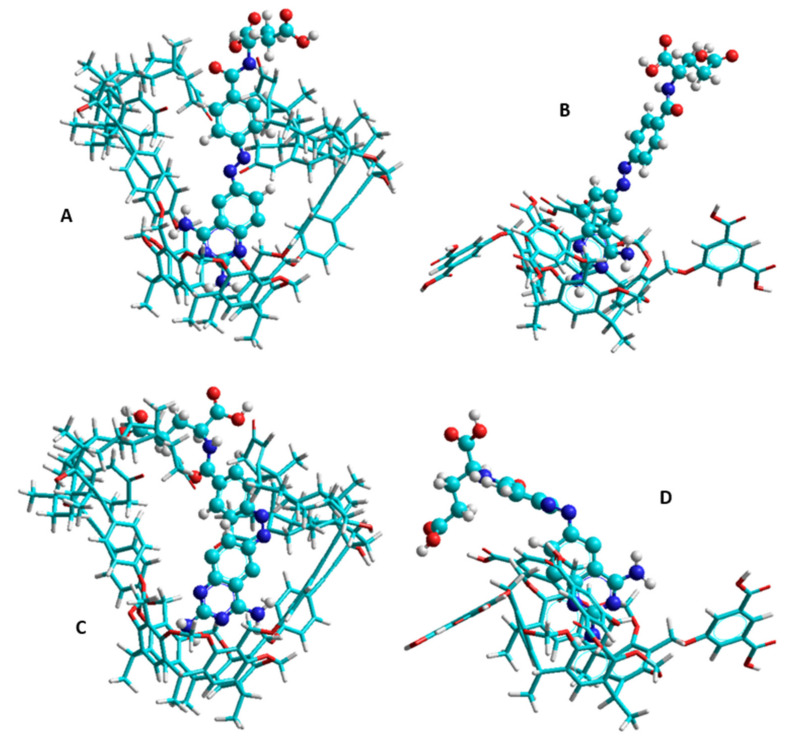
The 3D structures of the complexes of *trans*-PHX with TAC (**A**), *trans*-PHX with TDC (**B**), *cis*-PHX with TAC (**C**) and *cis*-PHX with TDC (**D**) calculated at AM1 level using the TIP3P solvation model. Solvent molecules are omitted due to the clarity.

The calculations, performed to determine the thermodynamic parameters associated with the complex formation of PHX with TAC and TDC molecules, used the explicit solvent model, therefore, motions of solvent molecules were also considered. Table 3 summarizes these results and Figure 6 represents the conformations of the most stable complexes. The agreement of the calculated thermodynamic values and the results of the experiments support the appropriateness of the applied model. Considering the conformations and the accurately determined translation–rotation–vibration terms of the entropy, the translational entropy of the solvent molecules is found to be up to 85 percent responsible for the total entropy gain during formation of PHX–TAC complexes. The contribution of the translational entropy remains high in the case of the formation of PHX-TDC complexes; the decreased vibrational entropy of the dicarboxylatophenoxy moieties reduces the entropy gain. 

## 3. Discussion

The thermodynamics of the interaction of TAC and TDC molecules with the two isomers of PHX confirm the formation of stable host–guest complexes within the temperature range between 293 K and 308 K in a dimethyl sulfoxide medium. However, the enthalpy and entropy changes associated with the molecular interaction of PHX with TAC and TDC suggest entirely different mechanisms at molecular level. Modelling studies draw attention to the importance of the reorganization and destruction of the solvation shell upon the molecular association, and of the structural properties of the host molecules as well. Accordingly, both the TAC and TDC molecules possess two rims, which are potentially able to bind to the guest molecules: the core skeleton of the cavitands can include guests of an appropriate size, and the functionalized upper rims can also be bound to the guests. In the case of TAC, the large androst-4-en-3-one-17α-ethinyl functions block the entrance of the cavitand cavity prior the interaction with the PHX. This property causes the energy cost of the opening procedure during the interaction, and it results in the positive enthalpy change. As a parallel effect, the removal of the solvent molecules from the solvation shell of the steroidal moieties also costs energy, thereby further increasing the enthalpy change, while the increased freedom of the guest molecules after the leaving of the solvation shell causes the large entropy gain and results in the positive entropy changes of the association process. Modelling studies highlight that about 85 percent of the entropy gain originated from the increased translational freedom of the solvent molecules. This property is more pronounced in the case of *cis-*PHX–TAC complexes. The positive enthalpy and entropy changes, finally, support the formation of stable PHX–TAC complexes, but, considering that the temperature dependence is determined by the enthalpy changes, the stability of the complexes with TAC host increases at higher temperature. In contrast, the interactions of the *cis*- and *trans*-PHX with the TDC molecules are associated with negative enthalpy and entropy changes. This is probably due to the smaller reorganization energies of the 1,3-dicarboxylato-5-phenoxy moieties located at the upper rim of the TDC molecule and also due to the fact that the destruction of the smaller sized solvation shell of the 1,3-dicarboxylato-5-phenoxy arms result less free dimethyl sulfoxide molecules upon the interaction with the PHX guests. It is to be mentioned here, that in this case the increased translational entropy of the solvent molecules cannot overcompensate the reduced entropy of the aforementioned dicarboxylatophenoxy moieties, which is originated from the reduced vibrational freedom of these arms upon complex formation. 

Considering the two significantly different complex formation procedures, we can conclude that the opposite temperature dependence of the complex stabilities associated to the formation of PHX-TAC and PHX-TDC complexes is originated from the different functional groups located at the upper rim of the host molecules. Furthermore, the fact that the increased freedom of the solvent molecules can be determinant in the association process is also because dimethylsulfoxide, as a nonprotic solvent, does not form clusters after leaving the solvation shell. Therefore, there is no such process that could decrease the entropy gain.

## 4. Materials and Methods

### 4.1. Synthesis of Tetrakis(3,5-dicarboxylatophenoxy)-cavitand (TDC, **3**)

**Figure 7:**
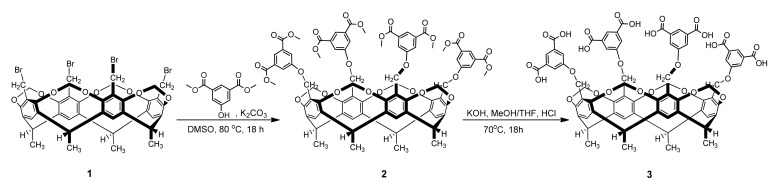
Schematic synthesis of TDC (**3**).

Dimethyl 5-hydroxyisophthalate (2.10 g, 10 mmol) and K_2_CO_3_ (2.07 g, 15 mmol) were dissolved in 50 mL of DMSO in a 100 mL round bottom flask under argon. The mixture was equipped with a magnetic stirrer and stirred for one hour at room temperature. Subsequently, Tetrabromocavitand **1** [22,23] (964.3 mg, 1.0 mmol) was added to the reaction mixture, the flask was stirred at 80 °C for 18 h under argon atmosphere. The mixture was cooled to room temperature and poured into 250 mL of 2% hydrochloric acid. The precipitate was filtered through a glass filter and washed with ice cold water and small portion of n-hexane, and dried under vacuum at 80 °C. Yield: 1.28 g/84%. The resulting product (Cavitand **2**) was immediately transferred into the following ester hydrolysis (Figure 7.).

Cavitand **2** (764.8 mg, 0.5 mmol) was dissolved in 20 mL of THF in a 100 mL round bottom flask, then 3 mL of Claisen’s alkali (prepared by dissolving 350 g of KOH in 250 cm^3^ of water, cooling and diluting to 1 L with MeOH) was added to the reaction mixture. The reaction mixture was refluxed at 70 °C for 18 h. The mixture was cooled to room temperature and the solution was acidified with 2 M hydrochloric acid. The precipitate was filtered through a glass filter and washed with ice cold water and small portion of n-hexane, and dried under vacuum at 80 °C. White powder (623.6 mg, 88%) was obtained (TDC, **3**). Melting point: >260 °C, δH (500.1 MHz, DMSO-d6): 1.89 (d, *J* = 7.4 Hz, 12H, C*H*_3_CH), 4.48 (d, *J* = 7.6 Hz, 4H, inner OC*H*_2_O), 4.85–4.94 (m, 12H, ArC*H*_2_O overlapping signals with CH_3_C*H*), 5.92 (d, *J* = 7.6 Hz, 4H, outer OC*H*_2_O), 7.03 (t, *J* = 7.5 Hz, 4H, Ar-*H*), 7.28 (dd, *J* = 7.6 Hz, 1.4 Hz, 4H, Ar-*H*), 7.49 (t, *J* = 7.5 Hz, 4H, Ar-*H*), 7.59 (dd, *J* = 7.6 Hz, 1.4 Hz, 4H, Ar-*H*), 7.89 (s, 4H, Ar-*H*), 10.62 (brs, 4H, COO*H*), 12.51 (brs, 4H, COO*H*), δC (125.1 MHz, DMSO-d6): 16.4, 30.6, 61.4, 100.0, 119.8, 122.7, 123.0, 125.4, 133.1, 139.6, 153.7, 159.1, 167.3.

### 4.2. Synthesis of PHX

**Figure 8:**
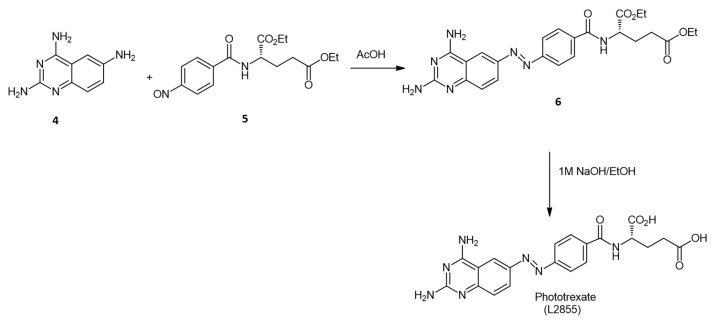
Schematic synthesis of PHX.

*Trans*-Phototrexate (*trans*-PHX) was synthesized in our institute. During the synthesis, we followed the procedure of Matera et al. [11], as shown in Figure 8. Briefly, **4** quinazoline-2,4,6-triamine was conjugated to **5** (S)-diethyl 2-(4-nitrosobenzamido)-pentanedioate to offer compound **6**. The latter was hydrolysed in mixture of sodium hydroxide and ethanol to yield PHX. 

**Figure 9:**
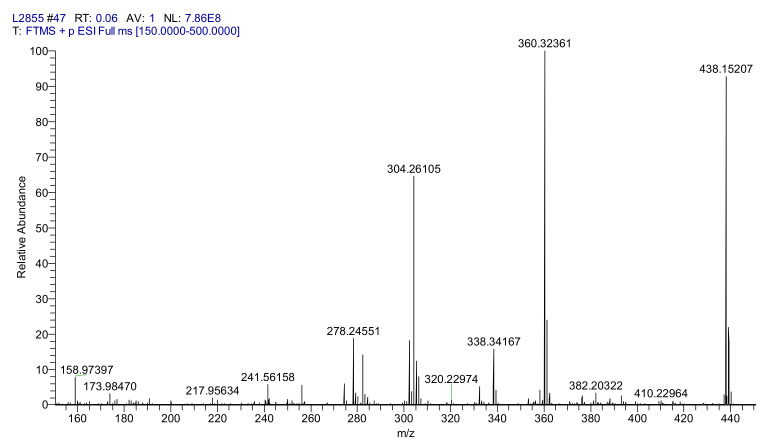
HRMS spectrum of PHX.

After chromatographic purification, the physicochemical data of PHX was in agreement with that of earlier published data. HRMS (ESI): m/z [M+H]^+^ calculated for C_30_H_30_N_7_O_5_^+^: 438.1520; found: 438.1520 (Figure 9.). 

### 4.3. Other Chemicals and Instruments

The applied solvent was dimethyl sulfoxide (DMSO) purchased from Merck (Darmstadt, Germany). 

Fluorimetric measurements were performed with a Fluorolog τ3 spectrofluorometer (Jobin-Yvon/SPEX, Longjumeau, France). Fluorescence spectra were recorded using 366 nm excitation wavelength. The emission values obtained at 490 nm were used for data evaluation. For data collection, photon counting method with 0.1 s integration time was used and 2 nm bandwidths set and quartz cuvettes with 1.0 cm thickness were applied.

The temperature dependence of binding constants was measured to determine the thermodynamic parameters associated to the binding of *trans*-PHX and *cis*-PHX to the cavitand derivatives (TAC and TDC). Accordingly, samples with different concentrations of the cavitand derivates (0–450 μM), and with constant concentration of *trans*- and *cis*-PHX (50 μM), were prepared and measured using 366 nm excitation wavelength at 293 K, 298 K, 303 K and 308 K temperatures.

The thermodynamically stable but pharmacologically inactive *trans*-PHX was isomerized with the application of UV-light (λ = 366 nm) provided by a Fluotest lamp (Original Hanau, Hanau, Germany). To ensure the complete isomerization, UV-vis spectra were recorded by a Specord Plus 210 spectrophotometer (Analytik Jena, Jena, Germany). 

### 4.4. Data Evaluation

Stability constants (*K*, dm^3^/mol) of PHX–cavitand complexes were calculated either using the Benesi–Hildebrand equation, assuming 1:1 complex stoichiometry:

(1) $$\frac{I_{0}}{I-I_{0}}=\frac{1}{A}+\frac{1}{A\cdot K\cdot \left[C\right]}$$

where *I_0_* and *I* are the fluorescence emission intensities of PHX in the absence and in the presence of the host, respectively; [*C*] is the molar concentration of the host molecule while A is a constant.

To determine the thermodynamic parameters, temperature dependence of the complex stabilities was examined, then the thermodynamic parameters were calculated using the Van ‘t Hoff equation:

(2) $$\ln K=\;-\frac{\Delta G}{R\cdot T}+\frac{\Delta S}{R}$$

where the ΔH and ΔS stand for the enthalpy and entropy changes of the complex formation, respectively, while ΔG is the Gibbs free energy change. *R* is the gas constant, while *T* is the temperature in Kelvin.

### 4.5. Modelling

Thermodynamic parameters of the Phototrexate–cavitand complexes were determined as follows. The enthalpy change was considered as the energy change calculated by subtracting the total energies of the reactants from the total energies of the products. Similarly, the entropy changes were calculated by subtracting the entropy terms of the reactants from the entropy terms of the products. Calculation of the entropy term was implemented in the HyperChem code as follows: after calculating the vibrational frequencies using the harmonic approximation, the entropy was then determined by the following equation:

(3) $$S_{vib}=R\underset{i}{\sum }\left\{\frac{\frac{h\nu_{i}}{kT}}{e^{\left(\frac{h\nu_{i}}{kT}\right)}-1}\right.\left.-\ln[1-e^{\left(-\frac{h\nu_{i}}{kT}\right)}]\right\}$$

Here, *ν_i_* is the frequency of vibration and *T* is the temperature. 

The molecular environment was considered by the TIP3P method while the simulation box randomly filled by the dimethyl sulfoxide molecules. The total energies of the species interacted have been calculated at semi-empirical AM1 level using HyperChem 8 code.

## 5. Conclusions

In this study, the complex formation thermodynamics of *trans*-PHX and *cis*-PHX was investigated with the host molecules TAC and TDC, both of which are cavitand derivatives. The applied methods were fluorescence spectroscopy and 3D modelling. Opposite temperature dependence of the stability of PHX-TAC and PHX-TDC complexes was found. These results imply different complex formation mechanisms.

The host–guest complex formation of PHX was described for the first time. PHX is a promising new molecule whose cytotoxic effect can be regulated with light. Complex formation can be another method that could make the physiological effect of this molecule more adjustable.

## Figures and Tables

**Table 1:** Stability constants of the investigated interactions determined at different temperatures.

Temperature (K)	log K			
	*trans*-PHX-TAC	*trans*-PHX-TDC	*cis*-PHX-TAC	*cis*-PHX-TDC
293	3.55 ± 0.19	3.05 ± 0.15	2.07 ± 0.17	3.08 ± 0.25
298	3.62 ± 0.21	2.95 ± 0.19	2.40 ± 0.21	3.03 ± 0.21
303	3.89 ± 0.18	2.88 ± 0.17	2.78 ± 0.22	2.90 ± 0.22
308	4.01 ± 0.20	2.73 ± 0.16	3.01 ± 0.20	2.88 ± 0.18

**Table 2:** Thermodynamic parameters of the complex formation between the cavitands and the isomers of PHX. ΔG values have been determined for 298 K.

	ΔH (kJ·mol^−1^)	ΔS (J·K^−1^·mol^−1^)	ΔG (kJ·mol^−1^)
*trans*-PHX-TAC	57.30 ± 2.84	262.97 ± 11.36	−21.10 ± 4.03
*trans*-PHX-TDC	−35.97 ± 3.69	−64.10 ± 10.43	−16.85 ± 3.21
*cis*-PHX-TAC	109.65 ± 6.93	414.12 ± 17.51	−13.83 ± 3.35
*cis*-PHX-TDC	−24.88 ± 3.20	−25.94 ± 4.72	−17.15 ± 3.62

**Table 3:** Thermodynamic parameters of the complex formation between the cavitands and the isomers of PHX determined at AM1 level applying the TIP3P solvation model. ΔG values have been calculated for 298 K.

	ΔH (kJ·mol^−1^)	ΔS (J·K^−1^·mol^−1^)	ΔG (kJ·mol^−1^)
*trans*-PHX-TAC	42.13	210.99	−20.89
*trans*-PHX-TDC	−33.63	−58.13	−16.29
*cis*-PHX-TAC	101.11	392.09	−15.79
*cis*-PHX-TDC	−23.54	−22.93	−16.70

## Data Availability

The data presented in this study are available on request from the corresponding author.
